# Liver T1 mapping in Fontan patients and patients with biventricular congenital heart disease – insights into the effects of venous congestions on diffuse liver disease

**DOI:** 10.1007/s10554-024-03314-5

**Published:** 2025-01-08

**Authors:** Yujiro Ide, Dominik Daniel Gabbert, Jan Hinnerk Hansen, Anselm Uebing, Inga Voges

**Affiliations:** 1 Department of Congenital Heart Disease and Pediatric Cardiology, University Hospital Schleswig-Holstein, Campus Kiel, Kiel, Germany; 2 German Center for Cardiovascular Research (DZHK), Partner site Hamburg/Lübeck/Kiel, Hamburg, Germany

**Keywords:** Liver mapping, Magnetic resonance imaging, Congenital heart disease, Fontan circulation

## Abstract

T1 relaxation time quantification on parametric maps is routinely used in cardiac imaging and may serve as a non-invasive biomarker for diffuse liver disease. In this study, we aimed to investigate the relationship between liver T1 values and cardiac function in patients with congenital heart disease (CHD) and compared patients with a biventricular circulation (BVC) to those with a Fontan circulation (FC). Magnetic resonance images from patients with CHD, obtained between June and December 2023 on a 1.5 T machine, were retrospectively reviewed. The examinations included cardiac cine sequences to assess ventricular mass and volumes, along with liver T1 mapping. T1 values were measured in eight liver segments and were compared with ventricular mass and volumes in patients with BVC and FC. In total, 104 patients (75 with BVC and 29 with FC) were included. T1 values varied significantly among the eight liver segments in both patient groups. In an age-matched comparison, patients with FC had significantly higher T1 values in all liver segments. In patients with BVC and right ventricular (RV) enlargement, a positive correlation between RV volume and T1 values in the right liver lobe was found (*R* > 0.504, *p* < 0.033). In patients with FC, the T1 values did not differ between patients with an extracardiac conduit or a lateral tunnel. Liver T1 mapping suggests more severe liver affection in patients with FC compared to those with BVC. It seems a valuable addition to cardiovascular magnetic resonance for patients who are at risk of systemic venous congestion.

## Introduction

In patients with congenital heart disease (CHD), chronic venous congestion can lead to liver disease [1]. The German National Register for CHD reports liver dysfunction as a major non-cardiac complication, observed in 6% of deceased patients with CHD [1]. Liver fibrosis has been noted in patients with a biventricular circulation (BVC) and within this group particularly in patients with tetralogy of Fallot (ToF) who have right ventricular (RV) dysfunction [2, 3]. However, in patients with a single ventricle (SV), liver injury is a ubiquitous sequela of the Fontan circulation (FC) known as Fontan-associated liver disease (FALD) [4, 5]. FALD is characterised by hepatic congestion and hepatic fibrosis progressing to cirrhosis caused by the absence of a ventricle supporting the pulmonary circulation. Besides hepatic fibrosis and cirrhosis, approximately 10% of patients diagnosed with FALD may develop hepatocellular carcinoma within 20 years [6]. Clinical symptoms are rare and late findings are common in patients with advanced disease, making early liver monitoring of important to detect alterations.

Liver biopsy is the gold standard for investigating histological liver abnormalities; however, it is invasive and typically samples only very confined liver regions. Therefore, non-invasive, repeatable and reliable monitoring techniques are necessary. Magnetic resonance (MR) mapping is a modern, reproducible technique that quantifies diffuse changes in organs like the liver, heart, and pancreas [7]. This imaging technique has a short history of application in the liver [8, 9], and relatively few reports of liver mapping, especially in patients with CHD, exist [10–12]. We hypothesised that this technique captures subclinical liver tissue alterations through numerical values, which relate to the cardiac status.

We therefore aimed to assess the liver status in patients with various CHDs using MR mapping and to correlate the findings with data obtained from cardiovascular MR imaging.

## Methods

### Ethical statement

Informed consent was obtained from all participants or their parents or guardians as appropriate. The study was approved by the ethics committee of University Hospital Schleswig-Holstein (D 588/24).

### Patients and study design

This retrospective study analysed MR images of patients with CHD who either had a BVC or FC. All patients are followed at the outpatient clinic of the University Hospital Schleswig-Holstein, and MR images were captured by a single examiner from June 2023 to December 2023.

### Magnetic resonance imaging

MR images were obtained with a 1.5-Tesla scanner (MAGNETOM Aera, Siemens Healthcare, Erlangen, Germany). Liver T1 mapping was performed in the axial plane at the liver’s widest dimension using a modified look-locker inversion sequence (echo time: 1.12 (1.08–1.20 ms); repetition time: 281 (272–361) ms; slice thickness: 8 (5–16) mm; flip angle: 35°; field of view: 360 × 307 mm^2^; matrix size: 256 × 218). Ventricular mass and volumes were measured using electrocardiographic-gated steady-state free precession cine images. All images were acquired during breath-holding.

### Analysis of magnetic resonance imaging data

**Fig. 1:**
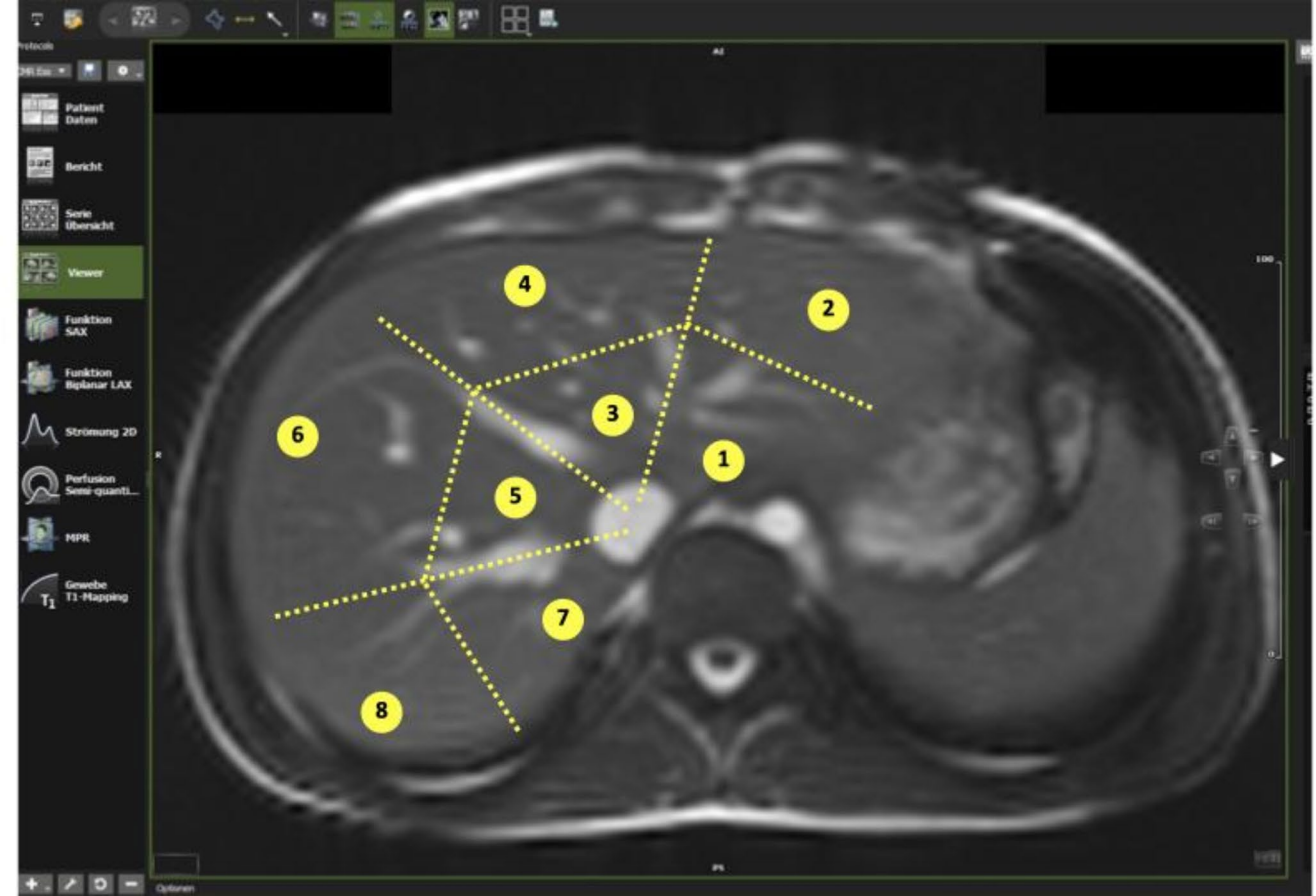
The figure shows the assessment of regional liver T1 values in eight defined liver segments. 1; centre, lateral, left lobe, 2; margin, lateral, left lobe, 3; centre, medial, left lobe, 4; margin, medial, left lobe, 5; centre, anterior, right lobe, 6; margin, anterior, right lobe, 7; centre, posterior, right lobe, 8; margin, posterior, right lobe

Acquired images were analysed using cvi42 (Circle, Cardiovascular Imaging, Calgary, Canada). Native liver T1 values were manually measured at eight different regions inspired by the Couinaud liver anatomy classification on axial images [13]. The liver segments were divided as follows: the right lobe into anterior and posterior sections and the left lobe into medial and lateral sections. Each section was further divided into regions adjacent to the inferior vena cava and close to the edge of the liver (Fig. 1). A circular region of interest of limited size (< 50 mm^2^) was placed in each liver segment, avoiding major blood vessels and bile ducts.

Cardiac parameters including end-diastolic volumes (EDV), end-systolic volumes (ESV), stroke volume (SV), end-diastolic myocardial mass (EDMM), and ejection fraction (EF) of both ventricles in patients with a BVC and of the single ventricle in patients with FC were measured from short-axis cine images.

Volumetry was performed as described before [14], and volumes and mass were indexed to body surface area (EDVi, ESVi, SVi, EDMMi) using the Mosteller formula. RV enlargement in patients with BVC was defined as EDV of the RV/ EDV of the left ventricle (LV) > 1.3” [15].

### Data analysis

Liver T1 values were compared between patients with BVC and patients with FC, and their association with cardiac parameters was examined. In patients with BVC, liver T1 values were compared based on the presence of the right ventricular enlargement. In patients with FC, liver T1 values ​​were analysed according to Fontan procedure type and dominance of the systemic ventricle. We also examined the association between liver T1 values ​​and cardiac parameters and the number of years since completion of the Fontan procedure. Furthermore, liver T1 values ​​between patients with BVC and those with FC were compared after age-matching.

### Statistical analysis

Continuous variables were presented as medians with ranges. The Mann–Whitney U test was used for non-normally distributed data, while the Chi-square test was used for categorical variables. The Friedman test compared median liver T1 values across eight liver segments within the same patient, with Bonferroni’s multiple comparisons applied for post hoc analysis. Correlations were assessed using univariate regression analysis with Spearman’s rank correlation. Statistical significance was set at *p* < 0.05. All statistical analyses were performed using EZR (Saitama Medical Center, Jichi Medical University, Saitama, Japan), a graphical user interface for R (The R Foundation for Statistical Computing, Vienna, Austria). More precisely, it is a modified version of R commander with added biostatistical functions [16].

## Results

### Patient demographics and baseline characteristics

In total, 104 patients (75 patients with BVC and 29 patients with FC) were included in the study. Patients with heterotaxy were excluded. In all BVC patients the anatomical right ventricle was the subpulmonary ventricle.

The median age was 22.4 (11.2–59.3) years for patients with BVC and 19.1 years (range 10.8–40.4) for patients with FC. Detailed data for each group is provided in Table 1.

**Table 1:** Patient characteristics

	FC		BVC	*p*-value
N	29		75	
Age (years)	19.1 (10.8–40.4)		22.4 (11.2–59.3)	0.016
BSA (m^2^)	1.6 (1.2–2.3)		1.9 (1.1–2.8)	0.001
Sex (F/M)	13/16		32/43	1.000
Years since Fontan completion	16.4 (7.2–26.7)		N/A	N/A
Type of Fontan operation	LT: 19 ECC: 10		N/A	N/A
Cardiac Diagnosis				
	dominant RV: 16	HLHS: 15	TOF: 16	N/A
		critical AS: 1	TGA: 9	N/A
	dominant LV: 11	DILV: 6	ASD/VSD/AVSD: 10	N/A
		TA: 3	AoV Disorder: 20	N/A
		PAIVS: 1	PuV Disorder: 3	N/A
		uAVSD: 1	isolated CoA: 4	N/A
	other: 2		other: 13	N/A
Systemic ventricle				
EDVi (ml/m^2^)	103 (53–262)		95 (59–185)	0.070
ESVi (ml/m^2^)	52 (19–209)		38 (19–90)	0.007
SVi (ml/m^2^)	53 (34–86)		53 (36–97)	0.928
EF (%)	54 (20–63)		58 (29–78)	0.001
EDMMi (g/m^2^)	42 (21–80)		54 (23–104)	0.005
Right ventricle				
EDVi (ml/m^2^)	N/A		96 (66–163)	N/A
ESVi (ml/m^2^)	N/A		42 (23–96)	N/A
SVi (ml/m^2^)	N/A		55 (41–99)	N/A
EF (%)	N/A		56 (41–70)	N/A
EDMMi (g/m^2^)	N/A		25 (15–38)	N/A
RV EDVi / LV EDVi ratio	N/A		1.0 (0.5–1.9)	N/A

*Abbreviations* ASD, atrial septal defect; uAVSD, unbalanced atrioventricular septal defect; VSD, ventricular septal defect; AoV, aortic valve; BSA, body surface area; BVC, biventricular circulation; cAS, critical aortic stenosis; DILV, double inlet left ventricle; ECC, extra-cardiac conduit; EDMMi, indexed end-diastolic myocardial mass; EDVi, indexed end-diastolic volume; EF, ejection fraction; ESVi, indexed end-systolic volume; FC, Fontan circulation; HLHS, hypoplastic left heart syndrome; LT, lateral tunnel; LV, left ventricle, N, number; N/A, not applicable; PAIVS, pulmonary atresia with intact ventricular septum; PuV, pulmonary Valve; RV, right ventricle; SVi, indexed stroke volume; TA, tricuspid atresia; TGA, transposition of the great arteries; TOF, tetralogy of Fallot

### Patients with BVC

**Fig. 2:**
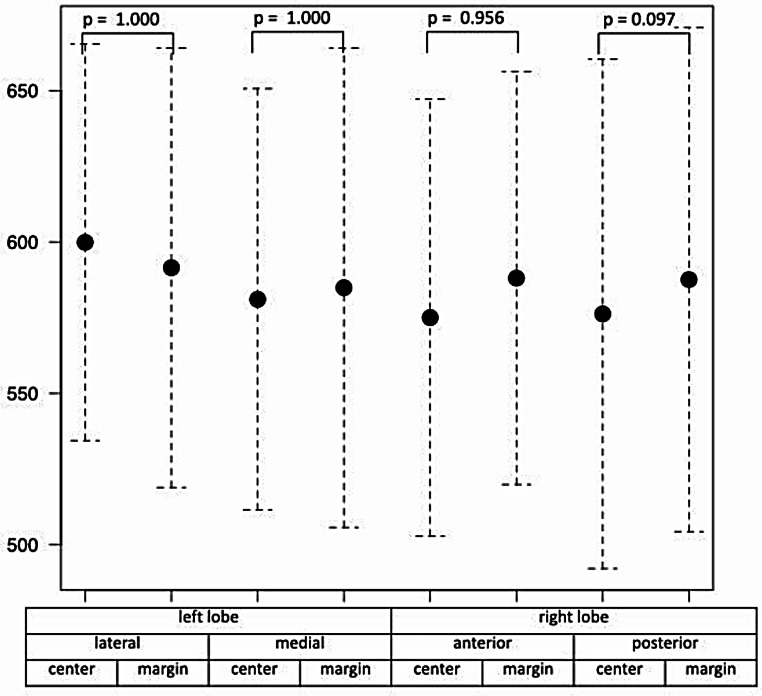
Comparison of T1 values in 75 patients with BVC in eight different segments of the liver

**Fig. 3:**
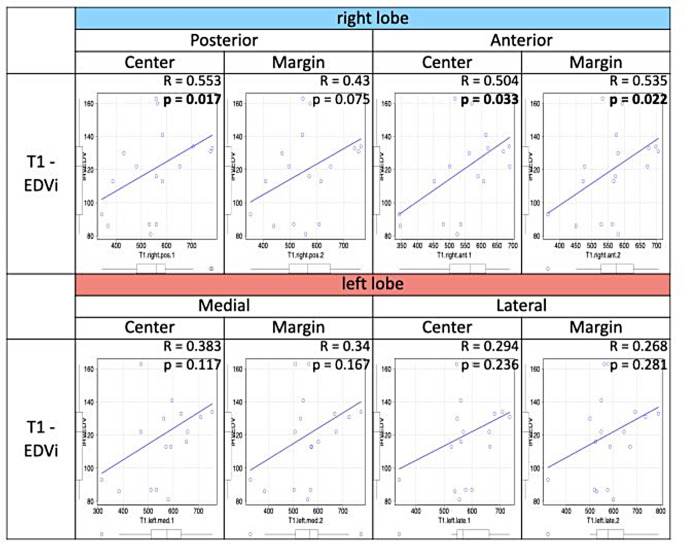
Correlation between the indexed end-diastolic volume of the RV and liver T1 value in 18 patients with dilated RV

In the 75 patients with BVC, liver T1 values did not correlate significantly with the RV parameters (EDVi, ESVi, SVi, EF, EDMMi; Table 2). Liver T1 values varied significantly among the eight liver areas (*p* = 0.002), but these values did not differ between regions near the inferior vena cava and marginal liver segments (Fig. 2). Liver T1 values did not differ significantly between patients with an enlarged RV (RVEDVi/LVEDVi ratio > 1.3) (*n* = 18) and others (*n* = 56) (Table 3); a moderate positive correlation was observed between RVEDVi and T1 values in the right liver lobe in patients presenting with an enlarged RV (Fig. 3).

**Table 2:** Correlations between cardiac MR data and liver T1 values in patients with BVC

	Right lobe				Left lobe			
	Posterior		Anterior		Medial		Lateral	
	center	margin	center	margin	center	margin	center	margin
EDVi	*R* = 0.081 *p* = 0.499	*R* = 0.035 *p* = 0.77	*R* = 0.108 *p* = 0.361	*R* = 0.012 *p* = 0.922	*R* = 0.016 *p* = 0.892	*R* = 0.100 *p* = 0.401	*R* = -0.012 *p* = 0.921	*R* = 0.035 *p* = 0.771
ESVi	*R* = 0.163 *p* = 0.172	*R* = 0.100 *p* = 0.406	*R* = 0.089 *p* = 0.454	*R* = 0.041 *p* = 0.73	*R* = 0.026 *p* = 0.827	*R* = 0.075 *p* = 0.527	*R* = 0.012 *p* = 0.917	*R* = 0.058 *p* = 0.628
SVi	*R* = 0.000 *p* = 0.997	*R* = -0.019 *p* = 0.877	*R* = 0.089 *p* = 0.455	*R* = -0.010 *p* = 0.935	*R* = -0.022 *p* = 0.851	*R* = 0.097 *p* = 0.416	*R* = -0.064 *p* = 0.593	*R* = -0.010 *p* = 0.931
EF	*R* = -0.179 *p* = 0.132	*R* = -0.13 *p* = 0.275	*R* = -0.078 *p* = 0.51	*R* = -0.068 *p* = 0.57	*R* = -0.096 *p* = 0.417	*R* = -0.025 *p* = 0.835	*R* = -0.081 *p* = 0.496	*R* = -0.046 *p* = 0.701
EDMMi	*R* = -0.062 *p* = 0.697	*R* = -0.122 *p* = 0.441	*R* = -0.121 *p* = 0.443	*R* = -0.242 *p* = 0.123	*R* = -0.304 *p* = 0.050	*R* = -0.204 *p* = 0.195	*R* = -0.226 *p* = 0.151	*R* = -0.175 *p* = 0.269

*Abbreviations* EDMMi, indexed end-diastolic myocardial mass; EDVi, indexed end-diastolic volume; EF, ejection fraction; EDMMi, indexed end-diastolic myocardial mass; ESVi, indexed end-systolic volume; SVi, indexed stroke volume

**Table 3:** Comparison between patients with enlarged RV and those without

	EDVi ratio < 1.3	EDVi ratio > 1.3	*p*-value
N	56	18	
Age	20.4 (11.2–59.3)	25.9 (13.1–49.9)	0.231
BSA	1.92 (1.08–2.80)	1.85 (1.37–2.14)	0.450
Pulmonic ventricle			
EDVi (ml/m^2^)	93 (66–145)	122 (81–163)	0.002
ESVi (ml/m^2^)	41 (23–66)	62 (36–96)	< 0.001
SVi (ml/m^2^)	54 (41–79)	62 (42–99)	0.231
EF (%)	58 (42–70)	50 (41–66)	0.001
EDMMi (g/m^2^)	24 (15–32)	29 (17–38)	0.009
RVEDVi / LVEDVi ratio	0.96 (0.45–1.24)	1.44 (1.30–1.85)	< 0.001
Liver T1 value			
Centre-Lateral-Left	593 (478–797)	569 (340–734)	0.229
Margin-Lateral-Left	588 (482–740)	576 (322–790)	0.552
Centre-Medial-Left	579 (509–713)	576 (315–755)	0.744
Margin-Medial-Left	577 (498–786)	566 (327–773)	0.271
Centre-Anterior-Right	580 (472–758)	567 (342–690)	0.334
Margin-Anterior-Right	589 (477–743)	574 (364–706)	0.274
Centre-Posterior-Right	569 (472–748)	560 (343–782)	0.447
Margin-Posterior-Right	578 (476–776)	566 (353–765)	0.447

*Abbreviations* BSA, body surface area; EDMMi, indexed end-diastolic myocardial mass; EDVi, indexed end-diastolic volume; EF, ejection fraction; EDMMi, indexed end-diastolic myocardial mass; ESVi, indexed end-systolic volume; LV, left ventricle; N, number; RV, right ventricle; SVi, indexed stroke volume

### Patients with FC

**Fig. 4:**
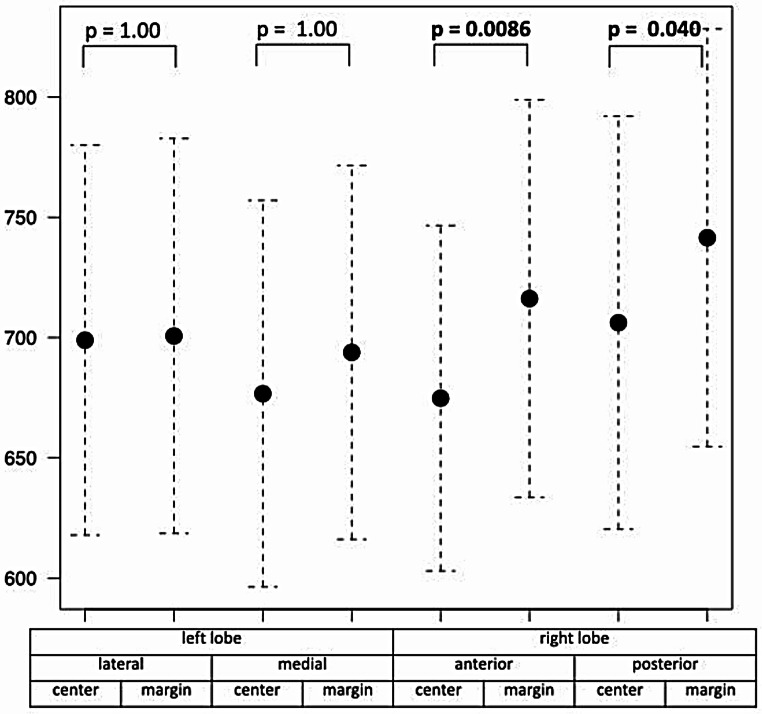
Comparison of T1 values in 29 patients with FC in eight different segments of the liver

**Fig. 5:**
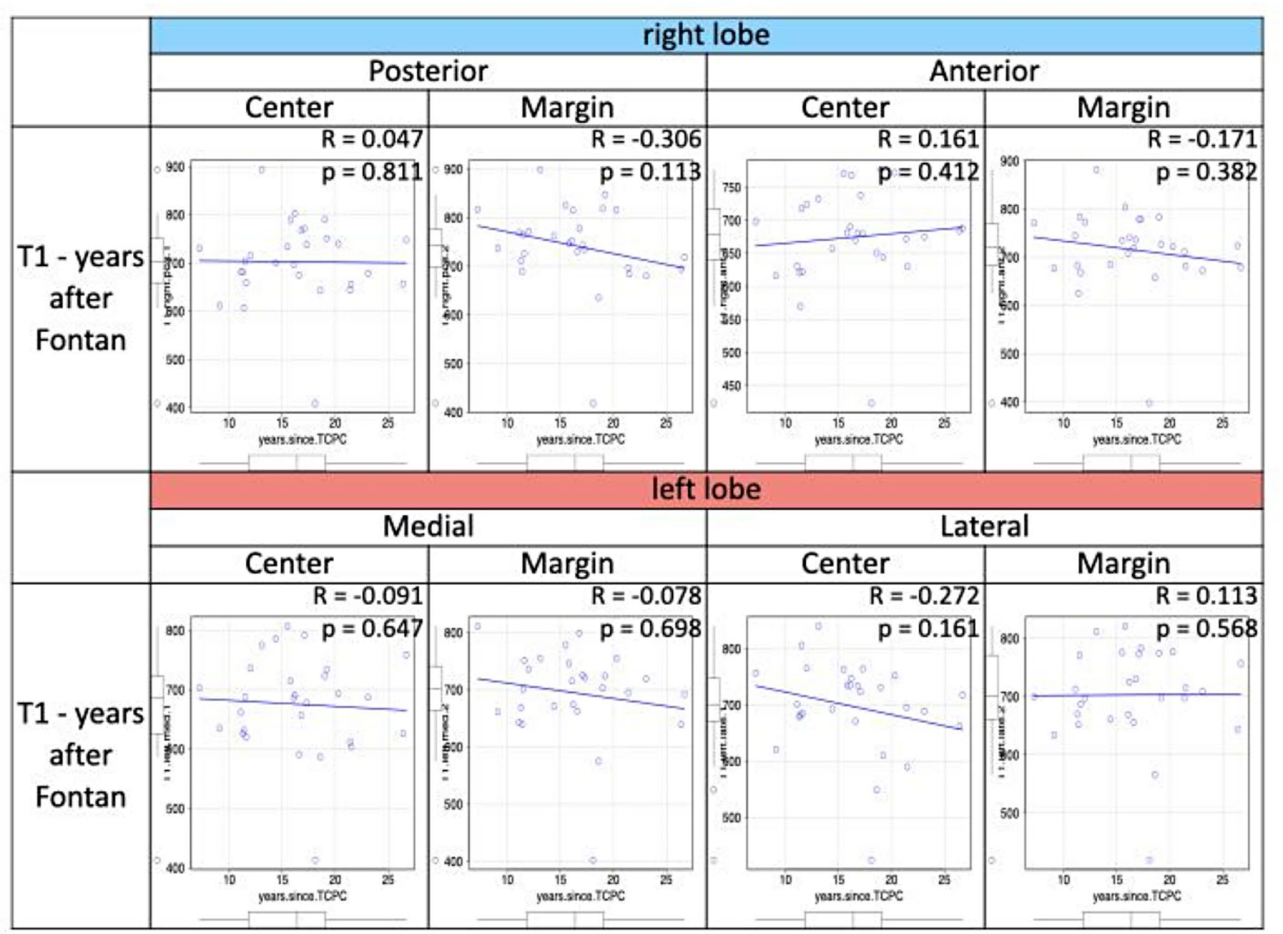
Correlation between T1 values in eight different segments of the liver and years after Fontan operation in 29 patients with FC

Liver T1 values differed significantly among the eight segments (*p* < 0.001), with significantly higher T1 values observed close to the inferior vena cava in the right lobe but not in the left lobe (Fig. 4). Patients with a right dominant ventricle (*n* = 16) had slightly larger ventricular volumes and lower EF than patients with a left dominant ventricle (*n* = 11), although no significant differences were observed in age, body surface area, years since Fontan completion, or the mode of Fontan operation. No significant differences in liver T1 values were found between these two groups (Table 4). Furthermore, T1 values did not differ between patients with an extracardiac conduit versus a lateral tunnel (*p* > 0.2 for all liver segments). No correlation was found between the years since Fontan operation and liver T1 values for all liver segments in the entire group of patients with FC (Fig. 5).

**Table 4:** Comparison between Fontan patients with dominant LV and those with dominant RV

	Dominant LV (*n* = 11)	Dominant RV (*n* = 16)	*p*-value
Age (years)	20.8 (13.6–40.4)	19.1 (13.3–27.4)	0.430
BSA (m^2^)	1.70 (1.52–2.25)	1.63 (1.29–2.10)	0.277
Sex (F/M)	6/5	6/10	0.452
Mode of Fontan operation; ECC / LT	5/6	4/12	0.411
Years after Fontan completion	15.7 (9.1–26.7)	16.7 (11.1–26.4)	0.635
Systemic ventricle			
EDVi (ml/m^2^)	98 (70–136)	112 (74–262)	0.098
ESVi (ml/m^2^)	41 (28–64)	57 (29–209)	0.088
SVi (ml/m^2^)	54 (39–76)	53 (42–86)	0.570
EF (%)	56 (47–61)	50 (20–63)	0.053
EDMMi (g/m^2^)	49 (27–65)	42 (33–80)	0.863
Liver T1 value			
Centre-Lateral-Left	718 (549–840)	691 (424–806)	0.537
Margin-Lateral-Left	698 (566–821)	704 (419–783)	0.570
Centre-Medial-Left	715 (587–807)	670 (413–786)	0.312
Margin-Medial-Left	725 (575–778)	674 (402–799)	0.154
Centre-Anterior-Right	681 (616–771)	673 (423–777)	0.444
Margin-Anterior-Right	711 (658–882)	722 (397–784)	0.882
Centre-Posterior-Right	734 (612–894)	689 (409–803)	0.521
Margin-Posterior-Right	737 (635–899)	743 (418–847)	0.921

*Abbreviations* BSA, body surface area; ECC, extracardiac conduit; EDMMi, indexed end-diastolic myocardial mass; EDVi, indexed end-diastolic volume; ESVi, indexed end-systolic volume; EF, ejection fraction; LT, lateral tunnel; LV, left ventricle; RV, right ventricle; SVi, indexed stroke volume

### Comparison between age-matched FC and BVC patients

**Fig. 6:**
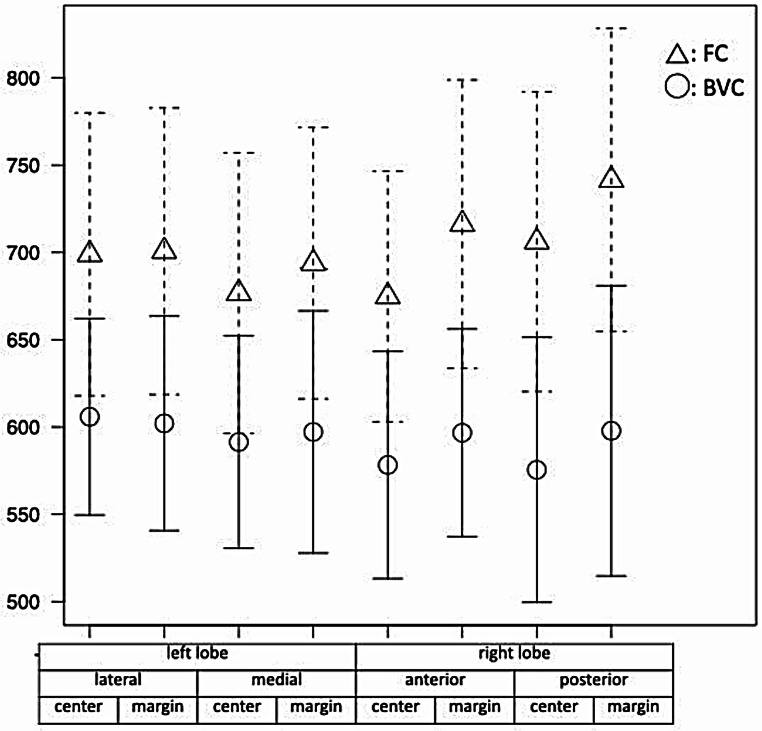
Comparison of T1 values in eight different liver segments among age-matched patients with FC and BVC

Twenty-nine age-matched patients from each patient group were selected. No significant differences were observed in the systemic ventricular volumes (EDVi, ESVi, and SVi) between these two groups; however, EF and EDMMi were markedly lower among patients with FC (*p* < 0.05, Table 5). Liver T1 values were significantly higher in patients with FC across all eight liver segments (*p* < 0.001) compared to those in the BVC group (Table 5; Fig. 6).

**Table 5:** Comparison between patients with FC and those with BVC

	FC (*n* = 29)	BVC (*n* = 29)	*p*-value
Sex (F/M)	13/16	11/18	0.790
Age (years)	19.1 (10.8–40.4)	19.4 (11.2–40.2)	0.938
BSA	1.64 (1.21–2.25)	1.80 (1.08–2.80)	0.068
Systemic ventricle			
EDVi (ml/m^2^)	103 (53–262)	100 (65–185)	0.911
ESVi (ml/m^2^)	52 (19–209)	46 (26–90)	0.453
SVi (ml/m^2^)	53 (34–86)	56 (36–97)	0.190
EF (%)	54 (20–63)	57 (29–65)	0.032
EDMMi (g/m^2^)	42 (21–80)	56 (40–93)	0.001
Liver T1 value			
Centre-Lateral-Left	701 (424–840)	595 (478–708)	< 0.001
Margin-Lateral-Left	700 (419–821)	588 (482–740)	< 0.001
Centre-Medial-Left	686 (413–807)	584 (471–755)	< 0.001
Margin-Medial-Left	702 (402–811)	575 (502–773)	< 0.001
Centre-Anterior-Right	679 (423–777)	570 (453–688)	< 0.001
Margin-Anterior-Right	722 (397–882)	589 (473–710)	< 0.001
Centre-Posterior-Right	703 (409–894)	573 (387–725)	< 0.001
Margin-Posterior-Right	743 (418–899)	595 (409–776)	< 0.001

*Abbreviations* BVH, biventricular heart; BSA, body surface area; BVC, biventricular circulation; EDMMi, indexed end-diastolic myocardial mass; EDVi, indexed end-diastolic volume; ESVi, indexed end-systolic volume; EF, ejection fraction; FC, Fontan circulation; F, Female; M, Male; SVi, indexed stroke volume

## Discussion

This study demonstrates that liver T1 values vary among different liver segments, regardless of ventricular physiology. A moderate positive correlation was found between RV volumes and liver T1 values in patients with enlarged RV. Median liver T1 values were higher in patients with FC compared with those of patients with BVC.

Chronic systemic venous congestion, particularly in patients with ventricular dysfunction or without a sub-pulmonary ventricle (FC patients), raises concerns about progressive liver injury [2–5]. The pathology of the liver begins with fibrosis, which progresses to cirrhosis that can promote the development of hepatocellular carcinoma [17]. Early detection of pathological liver changes is crucial, ideally with non-invasive, easily repeatable, and reliable imaging. In recent years, ultrasound elastography has been used as an imaging modality to assess liver fibrosis, but it has some limitations, such as measurement difficulties in patients with ascites and poor reproducibility of measurements in patients with obesity and those with fatty liver. However, MR imaging offers advantages, as it is normally not affected by these conditions and can be performed simultaneously with cardiovascular MR, making it highly relevant for patients with cardiac disease.

Liver T1 mapping is a relatively new method for assessing liver fibrosis [8, 9]. However, several studies have reported a strong correlation between liver T1 values and scoring systems for liver disease in the absence of congestive hepatopathy [18, 19]. Interestingly, even in healthy livers T1 values vary by segment [20].

For patients with CHD and a BVC, several reports of liver abnormalities exist, particularly in patients with ToF. These studies examined liver status in the setting of chronic venous congestion due to RV dysfunction [2–4]. Ravndal et al. used various imaging techniques and reported that 30% of patients with ToF had mild liver fibrosis [3]. In contrast, Kazour et al. reported no difference in liver T1 values between patients with ToF and healthy controls [4]. However, the link between liver abnormalities and the status of the RV was not sufficiently studied in these reports. In the current study, we focused on the relationship between RV size and function and liver T1 values. We compared liver T1 values between patients with and without RV enlargement and found no differences between the two groups in any part of the liver. However, in patients with RV enlargement, a moderate positive correlation between EDVi and liver T1 values was observed, particularly in the right liver lobe. Although no correlation was observed in the entire cohort of BVH patients, we speculate that the reason for the moderate correlation observed only in patients with RV enlargement is due to population bias, in that the majority of BVH patients (56 patients, 75%) did not have RV enlargement. In addition, the fact that the correlation between RVEDVi and liver T1 remained moderate even in patients with RV enlargement (median RV/LV EDV ratio = 1.44) suggests that the liver is not affected unless a patient has considerable RV enlargement. Notably, hepatocellular carcinoma in patients with ToF has predominantly been found in the right lobe, suggesting that the right lobe is more susceptible to venous congestion [21–23].

Several studies have reported that patients with FC have higher liver T1 values than healthy controls. In a previous study from our group that included 29 patients with FC, median T1 values were 735 (705–764) ms in the left lobe and 745 (715–784) ms in the right lobe [24]. Similar liver T1 values ​​have been reported in other studies that did not focus on the different liver lobes. Greidanus et al. reported median liver T1 values of 728 (714–744) ms in 20 Fontan patients [12], while Beigh et al. reported a mean T1 value of 727 ± 49 ms in 16 patients with a FC [25].

There is no absolute reference for liver T1 values, as they vary depending on the vendor of the MR machine and magnetic field strength [26, 27]. In our present study, using the same MR machine and post-processing software, the measured T1 values (700 ± 82 ms) were consistent with those described in the literature [12, 25], suggesting that liver T1 mapping is reproducible.

In previous studies, T1 values ​​were assessed at specific liver segments or averaged from several segments in the parasagittal view for patients with FC [11, 12, 25]. In our previous study, we mapped the entire liver using detailed measurements based on the Couinaud classification [24]. However, in our current study, we used a single axial image, which captures the widest area of ​​the liver, as a simple method for clinical practicality. Our results showed consistently higher liver T1 values ​​in all segments of patients with FC without exception compared with those of patients with BVC. Furthermore, our study confirms differences in liver T1 values between areas in the periphery of the IVC and the liver margins in patients with FC, especially in the right lobe, as reported by Beigh et al. [25]. This may imply that the peripheral right lobe is more susceptible to venous congestion, leading to faster development of liver fibrosis. If this speculation is correct, this could lead to an underestimation of fibrosis when measuring T1 values in different segments (e.g. near the centre of the left lobe). This should be kept in mind, especially when monitoring serial changes of liver T1 values in the same patient.

In the modern era, there remain two major types of Fontan procedures: the extracardiac conduit and the lateral tunnel. However, it remains controversial as to which procedure is superior [28]. Kisamori et al. reported that the extracardiac conduit resulted in worse liver outcomes compared with the lateral tunnel technique [29]. In our study, liver T1 values did not differ between the two surgical techniques. The follow-up period after Fontan surgery was, however, short (4.8 years). Weixler et al. reported no significant difference in liver complications between these procedures [30], supporting our results.

In addition, differences in ventricular physiology are always of interest when discussing the prognosis of patients with FC. Some studies discuss its association with prognosis in patients with FC, but the results remain controversial [31, 32]. In a previous study, we reported that the difference in ventricular physiology was not a risk factor for FALD [33], a finding supported by the present study. This result also aligns with the report of Beigh et al. [25].

Previously, we identified a longer period post-Fontan completion as a risk factor for advanced FALD [33]. Therefore, it was assumed that there would be a correlation between liver T1 values and years since Fontan completion, but no correlation was found for any segments of the liver in this study. Nevertheless, this result fits to another previous report from this group, which showed no differences in T1 values ​​among patients with FALD of different severity [24]. In contrast, Shiina et al. reported different results from ours, with a correlation between age and liver T1 in 16 patients with FC with an average age of 31 years [11]. This discrepancy may be due to the younger age of our patient cohort, which is, on average, more than 10 years younger.

## Conclusion

In this study, we investigated the association between liver T1 values and cardiac parameters in patients with CHD by accessing liver T1 values in each defined liver segment. Elevated liver T1 values were found in patients with possible systemic venous congestion, including patients with BVC and RV dilation as well as patients with a FC. Furthermore, liver T1 values also varied by segment. They were typically higher at the liver margins than at the centre, highlighting the importance of site measurement, especially when following the same patient longitudinally.

Liver T1 mapping can be a valuable addition to cardiac MRI studies for patients at risk of liver fibrosis due to potential systemic venous congestion.

## Data Availability

No datasets were generated or analysed during the current study.

## References

[1] Engelings CC, Helm PC, Abdul-Khaliq H et al (2016) Cause of death in adults with congenital heart disease - an analysis of the German National Register for congenital heart defects. Int J Cardiol 211:31–36 26970963 10.1016/j.ijcard.2016.02.133

[2] Yamamura K, Sakamoto I, Morihana E, Hirata Y, Nagata H, Yamasaki Y, Okumura Y, Kohashi K, Koto K, Tsutsui H, Ohga S (2019) Elevated non-invasive liver fibrosis markers and risk of liver carcinoma in adult patients after repair of tetralogy of Fallot. Int J Cardiol 287:121–126 31006598 10.1016/j.ijcard.2019.04.032

[3] Ravndal MEH, Borgwardt L, Juul K et al (2021) Liver fibrosis in patients with tetralogy of Fallot, an unrecognised complication? Cardiol Young 31(11):1796–1806 33719984 10.1017/S1047951121000901

[4] Kazour I, Serai SD, Xanthakos SA, Fleck RJ (2018) Using T1 mapping in cardiovascular magnetic resonance to assess congestive hepatopathy. Abdom Radiol (NY) 43(10):2679–2685 29500649 10.1007/s00261-018-1528-x PMC6120811

[5] Gordon-Walker TT, Bove K, Veldtman G (2019) Fontan-associated liver disease: a review. J Cardiol 74(3):223–232 30928109 10.1016/j.jjcc.2019.02.016

[6] Sagawa T, Kogiso T, Sugiyama H, Hashimoto E, Yamamoto M, Tokushige K (2020) Characteristics of hepatocellular carcinoma arising from Fontan-associated liver disease. Hepatol Res 50:853–862 32219953 10.1111/hepr.13500

[7] Banerjee R, Pavlides M, Tunnicliffe EM, Piechnik SK, Sarania N, Philips R, Collier JD, Booth JC, Schneider JE, Wang LM, Delaney DW, Fleming KA, Robson MD, Barnes E, Neubauer S (2014) Multiparametric magnetic resonance for the non-invasive diagnosis of liver disease. J Hepatol 60(1):69–77 24036007 10.1016/j.jhep.2013.09.002 PMC3865797

[8] Cassinotto C, Feldis M, Vergniol J, Mouries A, Cochet H, Lapuyade B, Hocquelet A, Juanola E, Foucher J, Laurent F, De Ledinghen V (2015) MR relaxometry in chronic liver diseases: comparison of T1 mapping, T2 mapping, and diffusion-weighted imaging for assessing cirrhosis diagnosis and severity. Eur J Radiol 84(8):1459–1465 26032126 10.1016/j.ejrad.2015.05.019

[9] Gilligan LA, Dillman JR, Tkach JA, Xanthakos SA, Gill JK, Trout AT (2019) Magnetic resonance imaging T1 relaxation times for the liver, pancreas and spleen in healthy children at 1.5 and 3 tesla. Pediatr Radiol 49(8):1018–1024 31049609 10.1007/s00247-019-04411-7

[10] de Lange C, Reichert MJE, Pagano JJ, Seed M, Yoo SJ, Broberg CS, Lam CZ, Grosse-Wortmann L (2019) Increased extracellular volume in the liver of pediatric Fontan patients. J Cardiovasc Magn Reson 21(1):39 31303178 10.1186/s12968-019-0545-4 PMC6628496

[11] Shiina Y, Inai K, Ohashi R, Nagao M (2021) Potential of liver T1 mapping for the detection of Fontan-associated liver disease in adults. Magn Reson Med Sci 20(3):295–302 32893257 10.2463/mrms.mp.2020-0063 PMC8424020

[12] Greidanus PG, Pagano JJ, Escudero CA, Thompson R, Tham EB (2023) Regional Elevation of Liver T1 in Fontan patients. CJC Pediatr Congenit Heart Dis 2(3):134–142 37969352 10.1016/j.cjcpc.2023.03.004 PMC10642140

[13] Couinaud C (1957) Le foie. In: Couinaud C (ed) Etudes Anatomiques et chirurgicales. Masson, Paris

[14] Schulz-Menger J, Bluemke DA, Bremerich J, Flamm SD, Fogel MA, Friedrich MG, Kim RJ, von Knobelsdorff-Brenkenhoff F, Kramer CM, Pennell DJ, Plein S, Nagel E (2020) Standardized image interpretation and post-processing in cardiovascular magnetic resonance – 2020 update: Society for Cardiovascular magnetic resonance (SCMR): Board of Trustees Task Force on standardized post-processing. J Cardiovasc Magn Reson. ;22(1) 10.1186/s12968-020-00610-6 PMC7066763 32160925

[15] Altmayer SP, Patel AR, Addetia K, Gomberg-Maitland M, Forfia PR, Han Y (2016) Cardiac MRI right ventricle / left ventricle (RV/LV) volume ratio improves detection of RV enlargement. J Magn Reson Imaging 43(6):1379–1385 26646199 10.1002/jmri.25110

[16] Kanda Y (2013) Investigation of the freely available easy-to-use software ‘EZR’ for medical statistics. Bone Marrow Transpl 48(3):452–458 10.1038/bmt.2012.244 PMC3590441 23208313

[17] Goldberg DJ, Surrey LF, Glatz AC, Dodds K, O’Byrne ML, Lin HC, Fogel M, Rome JJ, Rand EB, Russo P, Rychik J (2017) Hepatic fibrosis is Universal following Fontan Operation, and severity is Associated with Time from surgery: a liver biopsy and hemodynamic study. J Am Heart Assoc 6(5):e004809 28446492 10.1161/JAHA.116.004809 PMC5524062

[18] Breit HC, Block KT, Winkel DJ, Gehweiler JE, Henkel MJ, Weikert T, Stieltjes B, Boll DT, Heye TJ (2021) Evaluation of liver fibrosis and cirrhosis on the basis of quantitative T1 mapping: are acute inflammation, age and liver volume confounding factors? Eur J Radiol 141:109789 34051684 10.1016/j.ejrad.2021.109789

[19] Xu X, Zhu H, Li R, Lin H, Grimm R, Fu C, Yan F (2021) Whole-liver histogram and texture analysis on T1 maps improves the risk stratification of advanced fibrosis in NAFLD. Eur Radiol 31(3):1748–1759 32897416 10.1007/s00330-020-07235-4 PMC7880972

[20] Meloni A, Carnevale A, Gaio P, Positano V, Passantino C, Pepe A, Barison A, Todiere G, Grigoratos C, Novani G, Pistoia L, Giganti M, Cademartiri F, Cossu A (2024) Liver T1 and T2 mapping in a large cohort of healthy subjects: normal ranges and correlation with age and sex. MAGMA 37(1):93–100 38019376 10.1007/s10334-023-01135-6

[21] Augustyn A, Peng L, Singal AG, Yopp AC (2015) Surveillance for hepatocellular carcinoma secondary to cardiogenic cirrhosis in patients with congenital heart disease. Clin Res Cardiol 104(5):446–449 25875944 10.1007/s00392-015-0809-4 PMC4768748

[22] Happaerts S, Foucault A, Billiard JS, Nguyen B, Vandenbroucke-Menu F (2016) Combined hepatocellular-cholangiocarcinoma in a patient with Abernethy malformation and tetralogy of Fallot: a case report. Hepatology 64(5):1800–1802 27227347 10.1002/hep.28656

[23] Hyun Sung J, Sakamori R, Yamada R, Yoshioka T, Sakane S, Tahata Y, Shigekawa M, Kodama T, Hikita H, Tatsumi T, Takehara T (2022) Hepatocellular Carcinoma in a patient with tetralogy of Fallot: a Case Report and Literature Review. Intern Med 61(9):1361–1365 34670885 10.2169/internalmedicine.7827-21 PMC9152848

[24] Langguth P, Salehi Ravesh M, Moritz JD, Rinne K, Harneit PL, Khodami JK, Graessner J, Uebing A, Jansen O, Both M, Hansen JH (2022) Non-contrast enhanced magnetic resonance imaging for characterization of Fontan associated liver disease. Int J Cardiol 349:48–54 34808211 10.1016/j.ijcard.2021.11.048

[25] Beigh MVR, Pajunen KBE, Pagano JJ, Olugbuyi O, Harake DE, Noga ML, Tham EB (2023) T1 mapping of the myocardium and liver in the single ventricle population. Pediatr Radiol 53(6):1092–1099 36539566 10.1007/s00247-022-05560-y

[26] Dabir D, Child N, Kalra A, Rogers T, Gebker R, Jabbour A, Plein S, Yu CY, Otton J, Kidambi A, McDiarmid A, Broadbent D, Higgins DM, Schnackenburg B, Foote L, Cummins C, Nagel E, Puntmann VO (2014) Reference values for healthy human myocardium using a T1 mapping methodology: results from the International T1 multicenter cardiovascular magnetic resonance study. J Cardiovasc Magn Reson 16(1):69 25384607 10.1186/s12968-014-0069-x PMC4203908

[27] Oka H, Nakau K, Nakagawa S, Imanishi R, Shimada S, Mikami Y, Fukao K, Iwata K, Takahashi S (2023) Liver T1/T2 values with cardiac MRI during respiration. Cardiol Young 33(10):1859–1865 36281881 10.1017/S1047951122003274

[28] Daley M, d’Udekem Y (2021) The optimal Fontan operation: lateral tunnel or extracardiac conduit? J Thorac Cardiovasc Surg 162(6):1825–1834 33581907 10.1016/j.jtcvs.2020.11.179

[29] Kisamori E, Venna A, Chaudhry HE, Desai M, Tongut A, Mehta R, Clauss S, Yerebakan C, d’Udekem Y (2024) Alarmingrate of liver cirrhosis after the small conduit extracardiac Fontan: A comparative analysis with the lateral tunnel. J Thorac Cardiovasc Surg 168(4):1221-1227. 10.1016/j.jtcvs.2024.04.013 38688450

[30] Weixler VHM, Zurakowski D, Kheir J, Guariento A, Kaza AK, Baird CW, Del Nido PJ, Emani SM (2020) Fontan with lateral tunnel is associated with improved survival compared with extracardiac conduit. J Thorac Cardiovasc Surg 159(4):1480–1491e2 31928823 10.1016/j.jtcvs.2019.11.048

[31] Ghelani SJ, Colan SD, Azcue N, Keenan EM, Harrild DM, Powell AJ, Geva T, Rathod RH (2018) Impact of ventricular morphology on Fiber stress and strain in Fontan patients. Circ Cardiovasc Imaging 11(7):e006738 29970379 10.1161/CIRCIMAGING.117.006738

[32] Tweddell JS, Nersesian M, Mussatto KA, Nugent M, Simpson P, Mitchell ME, Ghanayem NS, Pelech AN, Marla R, Hoffman GM (2009) Fontan palliation in the modern era: factors impacting mortality and morbidity. Ann Thorac Surg 88:1291–1299 19766824 10.1016/j.athoracsur.2009.05.076

[33] Hansen JH, Khodami JK, Moritz JD, Rinne K, Voges I, Scheewe J, Kramer HH, Uebing A (2022 Summer) Surveillance of Fontan Associated Liver Disease in Childhood and Adolescence. Semin Thorac Cardiovasc Surg 34(2):642–650 10.1053/j.semtcvs.2021.04.005 33979666

